# Tumor endothelial cells accelerate tumor metastasis

**DOI:** 10.1111/cas.13336

**Published:** 2017-08-17

**Authors:** Nako Maishi, Kyoko Hida

**Affiliations:** ^1^ Vascular Biology Frontier Research Unit Institute for Genetic Medicine Hokkaido University Sapporo Japan

**Keywords:** Angiocrine factor, biglycan, endothelial cell, metastasis, stromal cell

## Abstract

Tumor metastasis is the main cause of cancer‐related death. Understanding the molecular mechanisms underlying tumor metastasis is crucial to control this fatal disease. Several molecular pathways orchestrate the complex biological cell events during a metastatic cascade. It is now well known that bidirectional interaction between tumor cells and their microenvironment, including tumor stroma, is important for tumor progression and metastasis. Tumor stromal cells, which acquire their specific characteristics in the tumor microenvironment, accelerate tumor malignancy. The formation of new blood vessels, termed as tumor angiogenesis, is a requirement for tumor progression. Tumor blood vessels supply nutrients and oxygen and also provide the route for metastasis. Tumor endothelial cells, which line tumor blood vessels, also exhibit several altered phenotypes compared with those of their normal counterparts. Recent studies have emphasized “angiocrine factors” that are released from tumor endothelial cells and promote tumor progression. During intravasation, tumor cells physically contact tumor endothelial cells and interact with them by juxtacrine and paracrine signaling. Recently, we observed that in highly metastatic tumors, tumor endothelial cells interact with tumor cells by secretion of a small leucine‐rich repeat proteoglycan known as biglycan. Biglycan from tumor endothelial cells stimulates the tumor cells to metastasize. In the present review, we highlight the role of tumor stromal cells, particularly endothelial cells, in the initial steps of tumor metastasis.

Cancer therapy, which includes surgical resection, chemotherapy, and radiotherapy to control primary tumors, has resulted in improved survival of cancer patients. However, tumor metastasis is still largely incurable and has become the primary cause of cancer‐related death.^1^ Hence, understanding the mechanism of metastasis is important to control metastatic diseases. The following complex cascade of events occurs during metastasis: Tumor cells invade the surrounding stroma and ECM and intravasate into the bloodstream, survive in the blood circulation with anoikis resistance, and then become disseminated through the circulation to reach distant organs, where they proceed to extravasate and invade into the parenchyma of distant tissues. These cells adapt to the new microenvironment and proliferate for metastatic colonization^2^ (Fig. 1).

**Figure 1:**
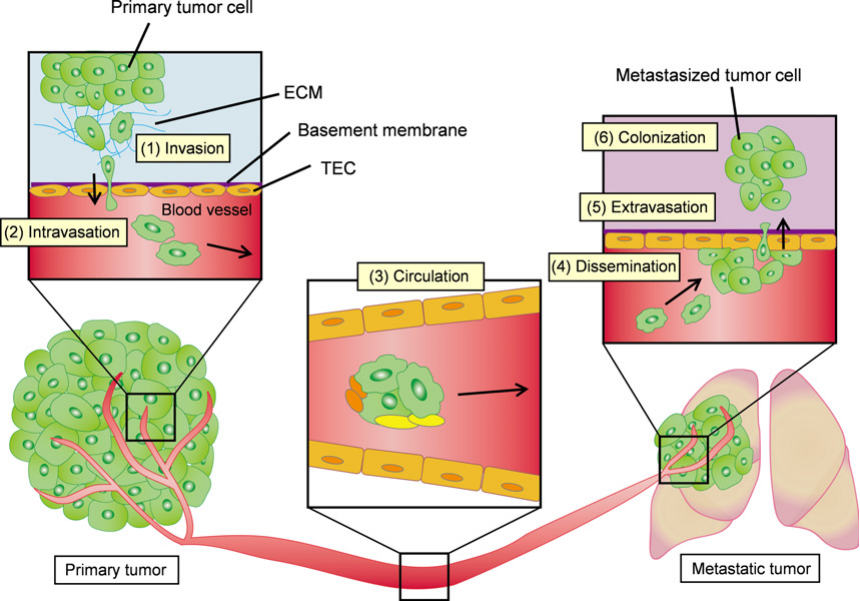
Steps in the development of tumor metastasis. (1) Tumor cells invade the surrounding stroma and extracellular matrix (ECM), (2) intravasate into the bloodstream, (3) survive in the circulation with anoikis resistance, (4) disseminate in the circulation and reach distant organs, (5) extravasate and invade into the parenchyma of distant tissues, (6) adapt to the new microenvironment and proliferate to form metastatic colonization. Stromal cells have important roles in metastasis dissemination. TEC, tumor endothelial cell.

Recently, researchers have studied the role of stromal cells in the tumor microenvironment. Stroma components include fibroblasts, macrophages, ECM, EC, lymphatic EC, and other inflammatory cells (Fig. 2). These non‐malignant stromal cells exhibit abnormal phenotypes compared with those in normal tissues. Tumor stromal cells are also known to secrete several protumorigenic factors to promote tumor growth and development.^3^ We studied TEC and observed several specific characteristics.^4^, ^5^ Because blood vessels surrounding tumors not only deliver nutrients and oxygen but also provide access for the tumor cells to metastasize, we assumed that TEC with abnormal phenotypes actively interact with tumor cells during tumor metastasis. We here summarize the current reports pertaining to tumor stromal cells in the tumor microenvironment. Furthermore, we highlight the role of TEC in promoting tumor metastasis.

**Figure 2:**
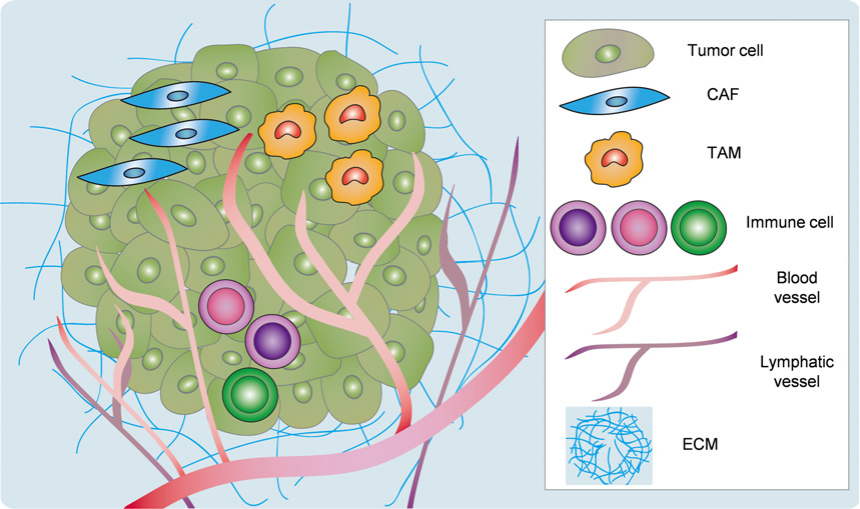
Tumor microenvironment is formed by tumor and stromal cells and extracellular matrix (ECM). CAF, cancer‐associated fibroblast; TAM, tumor‐associated macrophage.

## Role of Tumor Stromal Cells in Tumor Progression

It is well known that tumor progression and metastasis are not dependent on a tumor cell‐autonomous mechanism, but are controlled by the tumor microenvironment, including abnormal stromal cells.^6^ Fibroblasts are among the major components of tumor stroma.^7^ As tumors are considered wounds that do not heal,^8^ chronic tissue repair response occurs in tumor tissues, which causes tumor fibrosis. Activated fibroblasts in cancer are termed CAF, tumor‐associated fibroblasts, or activated myofibroblasts. Growth factors, such as TGF‐β, PDGF, and FGF, released by tumor cells are key mediators of CAF activation. CAF promoted tumorigenesis of tumor cells when cocultured with tumor cells, but not when cocultured with normal fibroblasts.^9^, ^10^ CAF activate the invasiveness of tumor cells with the production of MMP.^11^, ^12^, ^13^ CAF produce proinflammatory factors that activate NF‐κB signaling to promote tumor progression.^14^ Several recent studies have indicated that CAF remodel the tumor microenvironment and regulate tumor progression using their active secretome.^7^

TAM are also known to be important components of tumor stroma. Sources of TAM are tissue‐resident macrophage, bone marrow, or spleen. Several studies have revealed that TAM promote tumor progression and metastasis,^15^ although macrophages play an important role in the immune defense mechanism. Macrophages are classified as classically activated M1 and alternatively activated M2 macrophages.^16^ M1 macrophages produce proinflammatory cytokines and have antitumorigenic functions, whereas M2 macrophages promote anti‐inflammatory responses and have protumorigenic functions.^17^ However, it is unclear how macrophages switch from tumor‐suppressing to tumor‐promoting macrophages. TAM are proposed to share M2 characteristics and enhance tumor angiogenesis, resulting in tumor progression.^18^ TAM facilitate tumor cell intravasation through a paracrine signaling loop in which TAM‐derived EGF stimulates the invasiveness of tumor cells expressing the EGF receptor. Tumor cells, in turn, secrete CSF1 that attracts the CSF1 receptor expressing TAM.^19^, ^20^, ^21^ Invasion of tumor cells can be induced by other factors such as SDF1 (also known as CXCL12). Because SDF1 is synthesized by other stromal cells, including pericytes, fibroblasts, or EC^22^, ^23^ in the tumor microenvironment, cross‐talk between multiple stromal and tumor cells leads to tumor progression and metastasis.

## Abnormalities in Tumor Endothelial Cells

In 1971, Judah Folkman proposed that all tumors are angiogenesis dependent.^24^ Angiogenesis, defined as the growth of new blood vessels from the existing vasculature, is activated in response to the requirement for oxygen and nutrients.^25^ VEGF (known as vascular permeability factor) is one of the major molecules released from tumor cells to induce angiogenesis. Excessive VEGF in tumors causes vessel hyperpermeability and increases IFP, which results in chaotic vessel structure. Tumor blood vessel pattern is complex, non‐hierarchical, and disorganized.^26^, ^27^ TEC, which line tumor blood vessels, have generally been assumed to be genetically stable. However, several reports have indicated that TEC exhibit altered phenotypes compared to their normal counterparts.^28^ TEC have chromosomal abnormality^4^, ^29^, ^30^ and are resistant to anticancer drugs.^31^, ^32^, ^33^ TEC possess high proangiogenic properties, such as activated proliferation and migration, and upregulate several angiogenesis‐related genes.^34^, ^35^ In addition, TEC exhibit distinct gene expression patterns compared with those in normal EC.^34^, ^36^ Recently, some reports have suggested epigenetic alteration in TEC.^37^, ^38^, ^39^ These abnormalities in TEC point toward the need for understanding their role and function in tumor progression as well as of other stromal cells.

## Role of Tumor Endothelial Cells in Tumor Progression and Metastasis

Morphologically abnormal tumor vasculatures lead to tumor cell intravasation during tumor metastasis. VEGF–VEGF receptor signaling loosens the tight junctions that interconnect EC and, thereby, render the blood vessels permeable to leakage with high IFP. Because of high IFP and immature structure of tumor blood vessels lacking smooth muscles and pericytes, tumor cells easily permeate through tumor blood vessels.^40^ In addition to this passive entry route, TEC with abnormal phenotype, as described above, may also provide signals that actively promote tumor cells to metastasize.

Some researchers, including those in our group, have observed that TEC release specific growth factors, called angiocrine factors.^41^ Cao *et al*. revealed that FGF4, which is produced by B‐cell lymphoma cells, activate FGFR1 in the neighboring EC, and upregulate the Notch ligand Jagged1 in EC. In turn, Jagged1 on EC reciprocally induces Notch2–Hey1 in lymphoma cells.^42^ Cao *et al*. described that TEC convert indolent tumor cells to more aggressive cells with greater tumorigenicity, extranodal invasion, and chemoresistance.^42^, ^43^ Moreover, other groups have also demonstrated that the Notch signals in EC are involved in tumor progression and stem cell phenotype.^44^, ^45^, ^46^, ^47^ Notch activation in EC promotes tumor cell migration and metastasis along with neutrophil infiltration.^48^

In addition to the production of factors that simulate tumor cell progression, TEC also downregulate tumor suppressive factors. Slit2, which is negatively regulated by the endothelial EphA2 receptor, is one of the tumor suppressive angiocrine factors.^49^ The authors described that EphA2 represses Slit2 expression in EC to facilitate angiocrine‐mediated tumor invasion by blocking tumor suppressive signals.^49^ Such juxtacrine signals between EC and adjacent tumor cells could induce protumorigenic characteristics with increased expression of tumor‐stimulating factors or reduced expression of tumor‐suppressive factors.

CXCR7 on tumor vasculature is also involved in tumor progression and metastasis. CXCR7 is highly expressed in tumor blood vessels and is involved in tumor angiogenesis and growth.^22^, ^50^, ^51^ CXCR7 regulates CXCL12–CXCR4‐mediated tumor cell transendothelial migration.^52^ In contrast, another group demonstrated deletion of endothelial CXCR7 in conditional knockout mice, which resulted in an increased number of circulating tumor cells with elevated systemic CXCL12 levels.^53^ The authors suggested that CXCR7 partially functions as a scavenger receptor for CXCL12, and endothelial CXCR7 controls the amounts of systemic CXCL12.^53^ These findings suggest that tumor cell phenotype has plasticity and is determined by cues from TEC to confer tumors with aggressive and lethal properties.

Yadav *et al*.^54^ demonstrated the role of EC in protecting tumor cells from anoikis in the circulation. When tumor cells, particularly epithelial cells, detach from ECM in primary tumors, anoikis, which is defined as apoptosis induced by inadequate or inappropriate cell–matrix interaction, causes these cells to undergo cell‐cycle arrest and leads to rapid caspase‐mediated cell death. This is the reason why only few tumor cells reach the metastatic organs, although millions of these cells are shed from a tumor into circulation every day.^55^ However, the viable circulating EC have been reported to be observed in the blood of cancer patients.^56^, ^57^ These results led to the conclusion that activated EC expressing high levels of adhesion molecules can bind to tumor cells, protect them from anoikis in circulation, and facilitate their movement to distant organs.^54^

**Figure 3:**
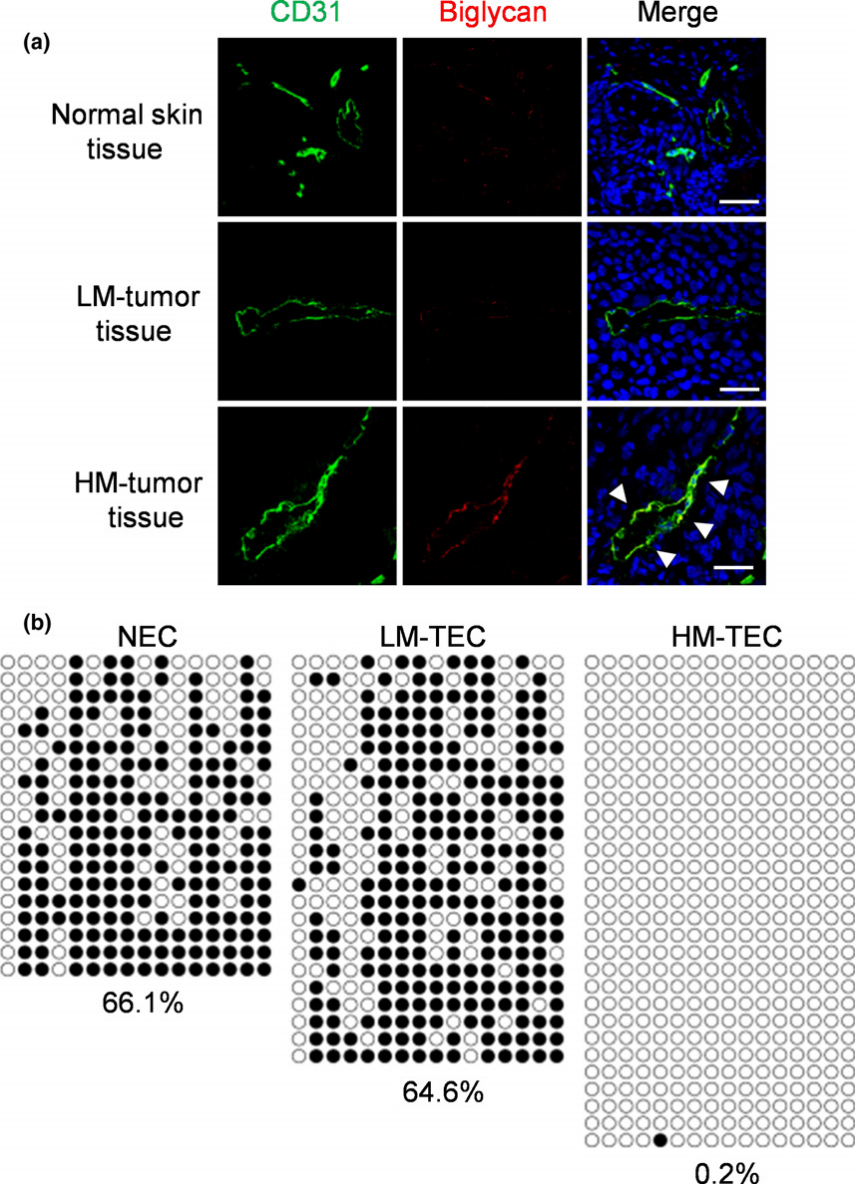
Biglycan is highly expressed in tumor endothelial cells (TEC) in highly metastatic tumors. (a) Biglycan expression in mouse normal skin tissues and indicated tumors dissected from mouse. (b) Bisulfite sequencing analysis of the biglycan promoter in endothelial cells (EC). White and black circles indicate unmethylated and methylated CpG dinucleotides, respectively. Reprinted from Maishi *et al*.,^39^ with permission from Sci Rep. HM‐TEC, highly metastatic tumor‐derived endothelial cell; LM‐TEC, low metastatic tumor‐derived endothelial cell; NEC, normal endothelial cell.

**Figure 4:**
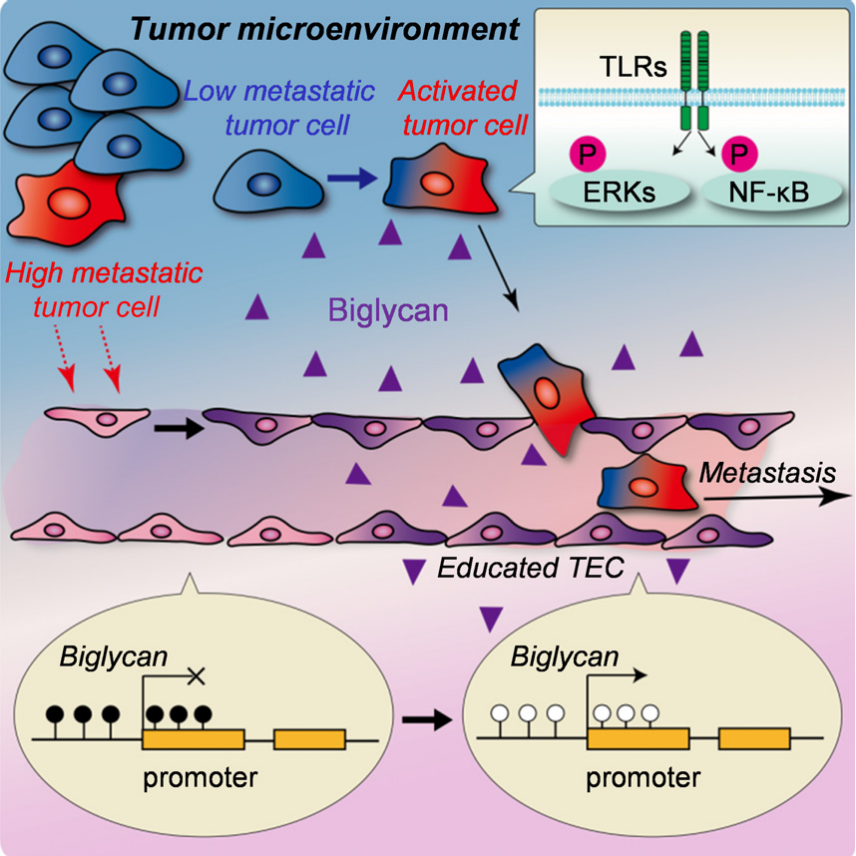
Promotion of tumor metastasis by tumor endothelial cells (TEC) through biglycan secretion. Tumor cells and the microenvironment develop TEC. In turn, these educated TEC express high levels of biglycan with epigenetic modification. TEC‐biglycan stimulates Toll‐like receptor (TLR)‐expressing tumor cells to metastasize by activation of nuclear factor‐kappa B (NF‐κB) and extracellular signal‐regulated kinase (ERK) signaling. Reprinted from Maishi *et al*.,^39^ with permission from Sci Rep.

Our group has reported the role of TEC in the initial steps of tumor metastasis.^39^ We have previously demonstrated the heterogeneity of TEC using two different types of TEC: HM‐TEC from highly metastatic and LM‐TEC from low metastatic tumors.^58^ HM‐TEC exhibited more proangiogenic phenotypes with the upregulation of angiogenesis‐related genes and more genetic instability with stem‐like phenotype and drug resistance, indicating that TEC acquire specific characteristics in response to their surrounding environment. Because tumor cells physically contact with TEC during tumor intravasation, we speculated that TEC, especially HM‐TEC with abnormal phenotypes, affect tumor cell behavior. *In vitro* data revealed that the ability of HM‐TEC to attract and adhere to tumor cells was greater than that of LM‐TEC or normal EC. Tumor cell transendothelial migration was enhanced on the HM‐TEC monolayer. Biglycan, a small leucine‐rich‐repeat proteoglycan, was specifically upregulated in HM‐TEC (Fig. 3a). Toll‐like receptor (TLR) 2 and TLR4 are reported as biglycan receptors, and TEC‐biglycan facilitated TLR‐expressing tumor cell migration through the activation of NF‐κB and ERK signaling. Biglycan knockdown in HM‐TEC decreased the number of circulating tumor cells and lung metastases *in vivo*. In addition, biglycan levels in the plasma of cancer patients were higher than those in healthy volunteers. Such high levels of biglycan were detected in the metastatic cases. The detailed molecular mechanisms by which tumor cells acquire their metastatic properties remain undefined, but our data indicated that TEC , which guard a gate for metastasis, provide a key molecule, biglycan, to allow tumor cells to break through the gate and results in hematogenous metastasis (Fig. 4). Interestingly, biglycan promoter in HM‐TEC had been markedly demethylated than in the other EC (Fig. 3b). It was also observed that the epigenetic modification that occurred in the tumor microenvironment was also involved in the increased expression of biglycan in TEC.^39^

Collectively, we and other groups identified an alternative role for TEC in facilitating tumor metastasis with orchestrating tumor cells and their microenvironment. Because EC in primary tumors are in close contact with tumor cells during intravasation, it is relevant that these have important roles in metastasis dissemination.

## Conclusions

Tumor stromal cells at primary sites are hijacked to support tumor development. Interaction between malignant tumor cells and their associated stromal cells in the tumor microenvironment is crucial for tumor progression. Tumor angiogenesis is regulated by several molecular signals and tumor‐derived factors. Neovascularized TEC function to transport nutrients and oxygen for tumor survival, growth, and metastatic spread. In addition to these functions, EC play an important role in the direct molecular regulation of tumor cell behavior. TEC express angiocrine factors to interact with tumor cells. Our study, as well as other recent studies, has proved the critical role of angiocrine signals in stimulating tumor progression and metastasis. EC can be reprogrammed to support tumor growth and metastasis with genetic and epigenetic alterations in the tumor microenvironment. Targeting EC–tumor cell interaction may be beneficial in developing anti‐metastasis approaches.

## Disclosure Statement

Authors declare no conflicts of interest for this article.

**Abbreviations**
CAF: cancer‐associated fibroblast
CSF1: colony‐stimulating factor 1
CXCL: C‐X‐C motif chemokine ligand
CXCR7: C‐X‐C chemokine receptor type 7
EC: endothelial cell
ECM: extracellular matrix
EGF: epidermal growth factor
ERK: extracellular signal‐regulated kinase
FGF: fibroblast growth factor
HM‐TEC: highly metastatic tumor‐derived endothelial cell
IFP: interstitial fluid pressure
LM‐TEC: low metastatic tumor‐derived endothelial cell
MMP: matrix metalloproteinase
NF‐κB: nuclear factor‐kappa B
PDGF: platelet‐derived growth factor
SDF1: stromal cell‐derived factor
TAM: tumor‐associated macrophage
TEC: tumor endothelial cell
TGF‐beta: transforming growth factor‐beta
TLR: Toll‐like receptor
VEGF: vascular endothelial growth factor

## References

[1] Gupta GP , Massague J . Cancer metastasis: building a framework. Cell 2006; 127: 679–95. 17110329 10.1016/j.cell.2006.11.001

[2] Fidler IJ . The pathogenesis of cancer metastasis: the ‘seed and soil’ hypothesis revisited. Nat Rev Cancer 2003; 3: 453–8. 12778135 10.1038/nrc1098

[3] Hanahan D , Coussens LM . Accessories to the crime: functions of cells recruited to the tumor microenvironment. Cancer Cell 2012; 21: 309–22. 22439926 10.1016/j.ccr.2012.02.022

[4] Hida K , Hida Y , Amin DN *et al* Tumor‐associated endothelial cells with cytogenetic abnormalities. Cancer Res 2004; 64: 8249–55. 15548691 10.1158/0008-5472.CAN-04-1567

[5] Hida K , Maishi N , Sakurai Y , Hida Y , Harashima H . Heterogeneity of tumor endothelial cells and drug delivery. Adv Drug Deliv Rev 2016; 99: 140–7. 26626622 10.1016/j.addr.2015.11.008

[6] Quail DF , Joyce JA . Microenvironmental regulation of tumor progression and metastasis. Nat Med 2013; 19: 1423–37. 24202395 10.1038/nm.3394 PMC3954707

[7] Kalluri R . The biology and function of fibroblasts in cancer. Nat Rev Cancer 2016; 16: 582–98. 27550820 10.1038/nrc.2016.73

[8] Dvorak HF . Tumors: wounds that do not heal. Similarities between tumor stroma generation and wound healing. N Engl J Med 1986; 315: 1650–9. 3537791 10.1056/NEJM198612253152606

[9] Olumi AF , Grossfeld GD , Hayward SW , Carroll PR , Tlsty TD , Cunha GR . Carcinoma‐associated fibroblasts direct tumor progression of initiated human prostatic epithelium. Cancer Res 1999; 59: 5002–11. 10519415 10.1186/bcr138 PMC3300837

[10] Orimo A , Gupta PB , Sgroi DC *et al* Stromal fibroblasts present in invasive human breast carcinomas promote tumor growth and angiogenesis through elevated SDF‐1/CXCL12 secretion. Cell 2005; 121: 335–48. 15882617 10.1016/j.cell.2005.02.034

[11] Stetler‐Stevenson WG , Aznavoorian S , Liotta LA . Tumor cell interactions with the extracellular matrix during invasion and metastasis. Annu Rev Cell Biol 1993; 9: 541–73. 8280471 10.1146/annurev.cb.09.110193.002545

[12] Sternlicht MD , Lochter A , Sympson CJ *et al* The stromal proteinase MMP3/stromelysin‐1 promotes mammary carcinogenesis. Cell 1999; 98: 137–46. 10428026 10.1016/s0092-8674(00)81009-0 PMC2853255

[13] Boire A , Covic L , Agarwal A , Jacques S , Sherifi S , Kuliopulos A . PAR1 is a matrix metalloprotease‐1 receptor that promotes invasion and tumorigenesis of breast cancer cells. Cell 2005; 120: 303–13. 15707890 10.1016/j.cell.2004.12.018

[14] Erez N , Truitt M , Olson P , Arron ST , Hanahan D . Cancer‐associated fibroblasts are activated in incipient neoplasia to orchestrate tumor‐promoting inflammation in an NF‐kappaB‐dependent manner. Cancer Cell 2010; 17: 135–47. 20138012 10.1016/j.ccr.2009.12.041

[15] Qian BZ , Pollard JW . Macrophage diversity enhances tumor progression and metastasis. Cell 2010; 141: 39–51. 20371344 10.1016/j.cell.2010.03.014 PMC4994190

[16] Mosser DM , Edwards JP . Exploring the full spectrum of macrophage activation. Nat Rev Immunol 2008; 8: 958–69. 19029990 10.1038/nri2448 PMC2724991

[17] Biswas SK , Mantovani A . Macrophage plasticity and interaction with lymphocyte subsets: cancer as a paradigm. Nat Immunol 2010; 11: 889–96. 20856220 10.1038/ni.1937

[18] Condeelis J , Pollard JW . Macrophages: obligate partners for tumor cell migration, invasion, and metastasis. Cell 2006; 124: 263–6. 16439202 10.1016/j.cell.2006.01.007

[19] Wyckoff J , Wang W , Lin EY *et al* A paracrine loop between tumor cells and macrophages is required for tumor cell migration in mammary tumors. Cancer Res 2004; 64: 7022–9. 15466195 10.1158/0008-5472.CAN-04-1449

[20] Goswami S , Sahai E , Wyckoff JB *et al* Macrophages promote the invasion of breast carcinoma cells via a colony‐stimulating factor‐1/epidermal growth factor paracrine loop. Cancer Res 2005; 65: 5278–83. 15958574 10.1158/0008-5472.CAN-04-1853

[21] Wyckoff JB , Wang Y , Lin EY *et al* Direct visualization of macrophage‐assisted tumor cell intravasation in mammary tumors. Cancer Res 2007; 67: 2649–56. 17363585 10.1158/0008-5472.CAN-06-1823

[22] Yamada K , Maishi N , Akiyama K *et al* CXCL12‐CXCR7 axis is important for tumor endothelial cell angiogenic property. Int J Cancer 2015; 137: 2825–36. 26100110 10.1002/ijc.29655

[23] Hayakawa Y , Ariyama H , Stancikova J *et al* Mist1 expressing gastric stem cells maintain the normal and neoplastic gastric epithelium and are supported by a perivascular stem cell niche. Cancer Cell 2015; 28: 800–14. 26585400 10.1016/j.ccell.2015.10.003 PMC4684751

[24] Folkman J . Tumor angiogenesis: therapeutic implications. N Engl J Med 1971; 285: 1182–6. 4938153 10.1056/NEJM197111182852108

[25] Hanahan D , Folkman J . Patterns and emerging mechanisms of the angiogenic switch during tumorigenesis. Cell 1996; 86: 353–64. 8756718 10.1016/s0092-8674(00)80108-7

[26] Pasqualini R , Arap W , McDonald DM . Probing the structural and molecular diversity of tumor vasculature. Trends Mol Med 2002; 8: 563–71. 12470989 10.1016/s1471-4914(02)02429-2

[27] McDonald DM , Choyke PL . Imaging of angiogenesis: from microscope to clinic. Nat Med 2003; 9: 713–25. 12778170 10.1038/nm0603-713

[28] Baluk P , Hashizume H , McDonald DM . Cellular abnormalities of blood vessels as targets in cancer. Curr Opin Genet Dev 2005; 15: 102–11. 15661540 10.1016/j.gde.2004.12.005

[29] Streubel B , Chott A , Huber D *et al* Lymphoma‐specific genetic aberrations in microvascular endothelial cells in B‐cell lymphomas. N Engl J Med 2004; 351: 250–9. 15254283 10.1056/NEJMoa033153

[30] Akino T , Hida K , Hida Y *et al* Cytogenetic abnormalities of tumor‐associated endothelial cells in human malignant tumors. Am J Pathol 2009; 175: 2657–67. 19875502 10.2353/ajpath.2009.090202 PMC2789618

[31] Akiyama K , Ohga N , Hida Y *et al* Tumor endothelial cells acquire drug resistance by MDR1 up‐regulation via VEGF signaling in tumor microenvironment. Am J Pathol 2012; 180: 1283–93. 22245726 10.1016/j.ajpath.2011.11.029

[32] Akiyama K , Maishi N , Ohga N *et al* Inhibition of multidrug transporter in tumor endothelial cells enhances antiangiogenic effects of low‐dose metronomic paclitaxel. Am J Pathol 2015; 185: 572–80. 25498238 10.1016/j.ajpath.2014.10.017

[33] Naito H , Wakabayashi T , Kidoya H *et al* Endothelial side population cells contribute to tumor angiogenesis and antiangiogenic drug resistance. Cancer Res 2016; 76: 3200–10. 27197162 10.1158/0008-5472.CAN-15-2998

[34] Matsuda K , Ohga N , Hida Y *et al* Isolated tumor endothelial cells maintain specific character during long‐term culture. Biochem Biophys Res Commun 2010; 394: 947–54. 20302845 10.1016/j.bbrc.2010.03.089

[35] Otsubo T , Hida Y , Ohga N *et al* Identification of novel targets for antiangiogenic therapy by comparing the gene expressions of tumor and normal endothelial cells. Cancer Sci 2014; 105: 560–7. 24602018 10.1111/cas.12394 PMC4317838

[36] St Croix B , Rago C , Velculescu V *et al* Genes expressed in human tumor endothelium. Science 2000; 289: 1197–202. 10947988 10.1126/science.289.5482.1197

[37] Deeb KK , Luo W , Karpf AR *et al* Differential vitamin D 24‐hydroxylase/CYP24A1 gene promoter methylation in endothelium from benign and malignant human prostate. Epigenetics 2011; 6: 994–1000. 21725204 10.4161/epi.6.8.16536 PMC3219083

[38] Luo W , Hu Q , Wang D *et al* Isolation and genome‐wide expression and methylation characterization of CD31+ cells from normal and malignant human prostate tissue. Oncotarget 2013; 4: 1472–83. 23978847 10.18632/oncotarget.1269 PMC3824530

[39] Maishi N , Ohba Y , Akiyama K *et al* Tumour endothelial cells in high metastatic tumours promote metastasis via epigenetic dysregulation of biglycan. Sci Rep 2016; 6: 28039. 27295191 10.1038/srep28039 PMC4904795

[40] Chang YS , di Tomaso E , McDonald DM , Jones R , Jain RK , Munn LL . Mosaic blood vessels in tumors: frequency of cancer cells in contact with flowing blood. Proc Natl Acad Sci USA 2000; 97: 14608–13. 11121063 10.1073/pnas.97.26.14608 PMC18966

[41] Butler JM , Kobayashi H , Rafii S . Instructive role of the vascular niche in promoting tumour growth and tissue repair by angiocrine factors. Nat Rev Cancer 2010; 10: 138–46. 20094048 10.1038/nrc2791 PMC2944775

[42] Cao Z , Ding BS , Guo P *et al* Angiocrine factors deployed by tumor vascular niche induce B cell lymphoma invasiveness and chemoresistance. Cancer Cell 2014; 25: 350–65. 24651014 10.1016/j.ccr.2014.02.005 PMC4017921

[43] Cao Z , Scandura JM , Inghirami GG , Shido K , Ding BS , Rafii S . Molecular checkpoint decisions made by subverted vascular niche transform indolent tumor cells into chemoresistant cancer stem cells. Cancer Cell 2017; 31: 110–26. 27989801 10.1016/j.ccell.2016.11.010 PMC5497495

[44] Zhu TS , Costello MA , Talsma CE *et al* Endothelial cells create a stem cell niche in glioblastoma by providing NOTCH ligands that nurture self‐renewal of cancer stem‐like cells. Cancer Res 2011; 71: 6061–72. 21788346 10.1158/0008-5472.CAN-10-4269 PMC3355476

[45] Lu J , Ye X , Fan F *et al* Endothelial cells promote the colorectal cancer stem cell phenotype through a soluble form of Jagged‐1. Cancer Cell 2013; 23: 171–85. 23375636 10.1016/j.ccr.2012.12.021 PMC3574187

[46] Ghiabi P , Jiang J , Pasquier J *et al* Endothelial cells provide a notch‐dependent pro‐tumoral niche for enhancing breast cancer survival, stemness and pro‐metastatic properties. PLoS One 2014; 9: e112424. 25380486 10.1371/journal.pone.0112424 PMC4224483

[47] Pedrosa AR , Trindade A , Carvalho C *et al* Endothelial Jagged1 promotes solid tumor growth through both pro‐angiogenic and angiocrine functions. Oncotarget 2015; 6: 24404–23. 26213336 10.18632/oncotarget.4380 PMC4695194

[48] Wieland E , Rodriguez‐Vita J , Liebler SS *et al* Endothelial Notch1 activity facilitates metastasis. Cancer Cell 2017; 31: 355–67. 28238683 10.1016/j.ccell.2017.01.007

[49] Brantley‐Sieders DM , Dunaway CM , Rao M *et al* Angiocrine factors modulate tumor proliferation and motility through EphA2 repression of Slit2 tumor suppressor function in endothelium. Cancer Res 2011; 71: 976–87. 21148069 10.1158/0008-5472.CAN-10-3396 PMC3032824

[50] Miao Z , Luker KE , Summers BC *et al* CXCR7 (RDC1) promotes breast and lung tumor growth in vivo and is expressed on tumor‐associated vasculature. Proc Natl Acad Sci USA 2007; 104: 15735–40. 17898181 10.1073/pnas.0610444104 PMC1994579

[51] Maishi N , Ohga N , Hida Y *et al* CXCR7: a novel tumor endothelial marker in renal cell carcinoma. Pathol Int 2012; 62: 309–17. 22524658 10.1111/j.1440-1827.2012.02792.x

[52] Zabel BA , Wang Y , Lewen S *et al* Elucidation of CXCR7‐mediated signaling events and inhibition of CXCR4‐mediated tumor cell transendothelial migration by CXCR7 ligands. J Immunol 2009; 183: 3204–11. 19641136 10.4049/jimmunol.0900269

[53] Stacer AC , Fenner J , Cavnar SP *et al* Endothelial CXCR7 regulates breast cancer metastasis. Oncogene 2016; 35: 1716–24. 26119946 10.1038/onc.2015.236 PMC4486335

[54] Yadav A , Kumar B , Yu JG , Old M , Teknos TN , Kumar P . Tumor‐associated endothelial cells promote tumor metastasis by chaperoning circulating tumor cells and protecting them from anoikis. PLoS One 2015; 10: e0141602. 26509633 10.1371/journal.pone.0141602 PMC4624958

[55] Butler TP , Gullino PM . Quantitation of cell shedding into efferent blood of mammary adenocarcinoma. Cancer Res 1975; 35: 512–6. 1090362

[56] Mancuso P , Burlini A , Pruneri G , Goldhirsch A , Martinelli G , Bertolini F . Resting and activated endothelial cells are increased in the peripheral blood of cancer patients. Blood 2001; 97: 3658–61. 11369666 10.1182/blood.v97.11.3658

[57] Beerepoot LV , Mehra N , Vermaat JS , Zonnenberg BA , Gebbink MF , Voest EE . Increased levels of viable circulating endothelial cells are an indicator of progressive disease in cancer patients. Ann Oncol 2004; 15: 139–45. 14679134 10.1093/annonc/mdh017

[58] Ohga N , Ishikawa S , Maishi N *et al* Heterogeneity of tumor endothelial cells: comparison between tumor endothelial cells isolated from high‐ and low‐metastatic tumors. Am J Pathol 2012; 180: 1294–307. 22245217 10.1016/j.ajpath.2011.11.035

